# *Campylobacter insulaenigrae* bacteremia with meningitis: a case report

**DOI:** 10.1186/s12879-021-06353-8

**Published:** 2021-07-01

**Authors:** Moe Kyotani, Tsuneaki Kenzaka, Hozuka Akita, Soichi Arakawa

**Affiliations:** 1 Department of Internal Medicine, Hyogo Prefectural Tamba Medical Center, 2002-7 Iso, Hikami-cho, Tamba, Hyogo 669-3495 Japan; 2 Division of Community Medicine and Career Development, Kobe University Graduate School of Medicine, 2-1-5, Arata-cho, Hyogo-ku, Kobe, Hyogo 652-0032 Japan; 3 Sanda City Hospital, 3-1-1, Keyaki-dai, Sanda, Hyogo 669-1326 Japan

**Keywords:** *Campylobacter insulaenigrae*, Meningitis, Bacteremia, Marine mammals, Meningeal irritation

## Abstract

**Background:**

The bacterium *Campylobacter insulaenigrae* was first isolated from marine mammals of Scotland in 2004. Only one case of *C. insulaenigrae* infection in humans has been previously reported.

**Case presentation:**

An 89-year-old Japanese man without dementia was admitted to our hospital, because he presented with a fever of 38 °C and weakness in right leg since 5 days. He had organized chronic subdural hematoma (CSH), and no history of pre-infection. At the time of admission, he had paralysis of the extraocular muscle, ataxia, and low manual muscle test score of the right side. He was suspected to have Miller Fisher syndrome; however, these symptoms improved without any treatment. On day 22 in the hospital, the patient presented a fever of 38.8 °C, left cranial nerve disorder, and hemiplegia. On day 25, the patient presented with signs of meningeal irritation; cerebrospinal fluid examination indicated an increase in the number of apocytes and a low glucose level. A contrast magnetic resonance imaging (MRI) scan of the patient’s head indicated a contrast enhancement effect in his right meninges. The blood culture showed presence of spirillums; 16S rRNA gene sequencing confirmed that the spirillums in the blood culture were *Campylobacter insulaenigrae* (*C. insulaenigrae*). We started treatment with meropenem for bacteremia and meningitis. When the symptoms improved, meropenem was replaced with ampicillin, based on the result of the drug sensitivity test. The treatment continued for 4 weeks.

**Conclusions:**

We report the first case of meningitis caused by *C. insulaenigrae* bacteremia in humans, and the second clinical report of *C. insulaenigrae* infection in humans. The bacterial strains isolated from humans and marine mammals had different genotypes. This suggests that different genotypes could be responsible for differences in the hosts. Further case studies are needed to establish the reasons behind the difference in the manifestations of *C. insulaenigrae* infections reported so far.

## Background

*Campylobacter insulaenigrae* was first isolated from the rectal swab of marine mammals (seals and a porpoise) of Scotland by Foster et al. in 2004 [1]. Later on, the bacterium was isolated from northern elephant seals in California [2] and the south American sea lion in Chile [3]. *C. insulaenigrae* is phylogenetically related to *C. jejuni*, *C. coli*, and *C. lari* [1]. The first case of infection caused by *C. insulaenigrae* in humans was reported by Chua K et al. in 2007 [4]. We report a case of *C. insulaenigrae* bacteremia with meningitis, as the second clinical case of *C. insulaenigrae* infection in humans.

## Case presentation

An 89-year-old Japanese man without dementia presented with a fever of 38 °C and weakness in right leg since 5 days. His symptoms showed no improvement; and therefore, he was admitted to our hospital. The patient reported no interaction with marine mammals (e.g. going to the aquarium, a household pet), no history of previous infection caused by them, and no event of eating raw fish.

The patient had a medical history of hypertension, glaucoma, and organized chronic subdural hematoma (CSH), which was treated with craterization at the age of 80-years. He had been prescribed the following medication: valsartan, 80 mg/day; valproic acid, 800 mg/day; magnesium oxide, 750 mg/day; pantosin, 3.0 g/day; and triazolam, 0.25 mg/day.

At the time of admission, the patient was conscious and lucid, and his vital signs were as follows: heart rate, 79 beats/min and regular; body temperature, 38.0 °C blood pressure, 157/86 mmHg; respiratory rate, 16 breaths/min; peripheral oxygen saturation, 95% in room air. Physical test results showed paralysis of extraocular muscle at downward and left gaze, ataxia, and low manual muscle test (MMT) score of the right side. No meningeal irritation signs (jolt accentuation, neck stiffness, Kernig’s sign, and Brudzinski’s sign) were observed at the time of admission. Two sets of blood culture on day 2 were sterile, and cerebrospinal fluid (CSF) could not be obtained during admission despite all efforts. Head computed tomography (CT) scans and magnetic resonance imaging (MRI) scans were unremarkable, except for the presence of organized CSH. Based on these findings and consultation with the neurologist, we suspected that the patient had Miller Fisher syndrome. However, the anti-ganglioside antibody test result was negative.

**Fig. 1:**
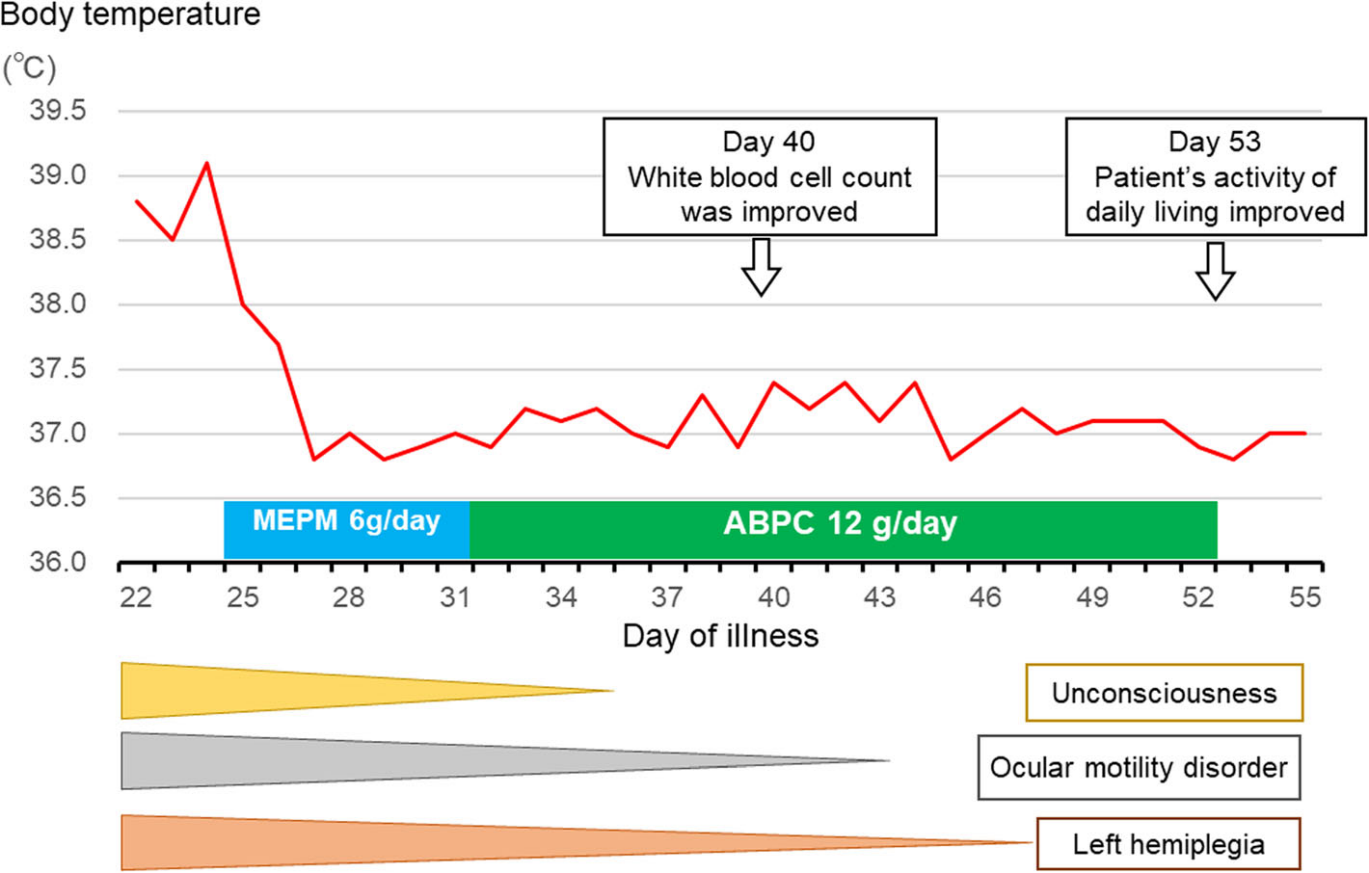
Clinical course after starting treatment. MEPM, meropenem; ABPC, ampicillin

After admission, the fever and neurological symptoms of the patient improved without treatment. He was discharged from the hospital and he stayed overnight at his home from day 21 to 22; he did not come into contact with saltwater or marine mammals and did not eat raw fish during his stay at home. He returned to the hospital on day 22 and presented with a fever of 38.8 °C and left hemiplegia with unconsciousness. Laboratory investigation results on day 24 were as follows (Table 1): white blood cell (WBC) count, 8610/μL (neutrophils, 84.8%; lymphocytes, 9.3%; and monocytes, 5.8%); and C-reactive protein (CRP), 19.42 mg/dL. Neurological findings on day 25 included positive meningeal irritation signs (Jolt accentuation and neck stiffness), ocular motility disorder in all directions, left cranial nerve disorder, low MMT score of left upper and lower limb, and extrapyramidal disorder. CSF examination results on the same day were as follows: initial pressure, 27 cmH_2_O; number of cells, 69/μL (number of monocytes, 13/μL; and number of apocytes 56/μL); protein, 168 mg/dL; and glucose level, 39 mg/dL (blood glucose level, 122 mg/dL). A phase-contrast MRI of the patient’s head indicated a contrast enhancement effect in his right meninges and cerebral edema in the frontal and parietal lobes, and blood culture reports showed the presence of spirillums. These results suggested bacteremia and meningitis. Taking into consideration the pharmacodynamics of antibiotics in CSF, we prescribed the patient with meropenem (2 g every 8 h) from day 24. The symptoms and laboratory findings improved after starting the treatment (Fig. 1).

**Table 1:** Laboratory investigation results of the patient on day 24

Parameter	Recorded value	Standard value
White blood cell count	8610/μL	4700–8700/μL
Neutrophils	84.8%	42–72%
Lymphocyte	9.3%	18–50%
Monocyte	5.8%	1–8%
Hemoglobin	12.5 g/dL	13–17 g/dL
Hematocrit	36.3%	40–50%
Platelet count	11.3 × 10^4^/μL	15–35 × 10^4^/μL
C-reactive protein	19.42 mg/dL	≤0.3 mg/dL
Total protein	6.3 g/dL	6.7–8.3 g/dL
Albumin	2.6 g/dL	3.9–4.9 g/dL
Total bilirubin	1.4 mg/dL	0.2–1.2 mg/dL
Aspartate aminotransferase	21 U/L	8–38 U/L
Alanine aminotransferase	15 U/L	4–44 U/L
Lactate dehydrogenase	158 U/L	120–230 U/L
Creatine phosphokinase	244 U/L	62–287 U/L
Blood urea nitrogen	27.0 mg/dL	8.5–20 mg/dL
Creatinine	0.76 mg/dL	0.53–1.02 mg/dL
Sodium	131 mEq/L	134–147 mEq/L
Potassium	3.9 mEq/L	3.5–5.0 mEq/L
Chloride	98 mEq/L	98–108 mEq/L
Glucose	187 mg/dL	70–109 mg/dL

The patient presented with fever and he was re-hospitalized on day 24. These are the laboratory investigation results during the re-admission

Blood culture results on day 22 showed the presence of gram-negative spirillums belonging to the genus *Campylobacter*; however, the species of the bacteria was not confirmed. 16S rRNA gene sequencing of the bacteria obtained from the blood culture identified the bacterial strain as *Campylobacter insulaenigrae*. The number of cells (monocytes and apocytes) and glucose levels in CSF samples were a clear indicator of bacterial meningitis; however, the culture and genetic analyses showed that the CSF samples were sterile. We changed the treatment from meropenem to ampicillin (2 g every 4 h) from day 32, based on the result of the drug sensitivity test using the disk method (Table **2**) and discontinued the treatment on day 53 (total duration 29 days); we observed that the day-to-day activity level of the patient reached levels similar to that before contracting bacteremia.

**Table 2:** Results of drug sensitivity test for *Campylobacter insulaenigrae* cultured on day 22

Disk method			Etest	
Antibiotic	Zone (mm)		Antibiotic	MIC (μg /ml))
Ampicillin	33	S	Ampicillin	8
Cefotaxime	> 30	S	Ceftriaxone	0.25
Ceftriaxone	8	R	Imipenem	0.125
Cefaclor	24	S	Meropenem	0.003
Meropenem	> 30	S	Minocycline	< 0.016
Erythromycin	> 30	S	Sulfamethoxazole-Trimethoprim	> 32
Fosfomycin	0	R	Cefepime	1.0
Levofloxacin	10	R	Clindamycin	0.064
			Ciprofloxacin	2
			Levofloxacin	2
			Tazobactam/Piperacillin	< 0.016

The “S” and “R” represent “susceptible” and “resistant”, respectively. The result of the Etest was obtained after the treatment. The assignment of S or R was according to the standard values for Campylobacter, according to the Clinical and Laboratory Standard Institute (*CLSI*). The assessment of the Etest was difficult, as a standard was not specified by the CLSI

## Discussion and conclusion

This case report presents a case of *C. insulaenigrae* bacteremia with meningitis. To the best of our knowledge, this is the first report of meningitis caused by *C. insulaenigrae* in humans, and the second report of infection by *C. insulaenigrae* in humans.

*C. insulaenigrae* was first isolated from marine mammals [1]; however, the patient in this study reported no contact with any marine mammals. We enquired about eating raw fish; however, we could not verify the history of contact with marine mammals. Therefore, the portal of entry was uncertain in this case, similar to the report by Chua K et al. [4] (Table **3**).

**Table 3:** Cases of *Campylobacter insulaenigrae* infection in humans

	Chua K et al. (2007) [4]	Current Study (2015)
Country	Australia	Japan
Age, Sex	60-year-old woman	89-year-old man
Symptoms	Fever, abdominal pain, and diarrhea	Fever and weakness in legs
Infection site	Intestinal canal, blood	meninges, blood
Medical history	Hemodialysis for autosomal dominant adult polycystic kidney and liver disease	Organized chronic subdural hematoma and hypertension
Interaction with marine mammals	None	None
Antibiotics for treatment	Ciprofloxacin, Azithromycin, and Meropenem	Meropenem and Ampicillin
Duration of therapy	24 weeks	4 weeks
GenBank	EF433401	DQ174183

The table compares the characteristics in the current study and the previous study reported in 2007 [4]. In both cases, there is no interaction with marine mammals and the portal of entry was uncertain in both of them

The first case of infection due to *C. insulaenigrae* was reported by Chua K et al. in 2007 [4]; the patient, a 60-year-old woman under hemodialysis, for treating autosomal dominant adult polycystic kidney, presented with gastroenteritis diagnosed by fever, diarrhea, and stomachache.

In this study, the CSF culture did not show the presence of *C. insulaenigrae*; however, the low glucose levels in the CSF suggested bacterial meningitis. In addition, we could not identify any other sites in the patient that were affected by bacteremia. Therefore, we clinically diagnosed the patient with meningitis caused by *C. insulaenigrae*. The organized CSH had not changed after craterization (done 9 years ago); and therefore, the patient’s neurological findings could have been induced by transient cerebral edema and meningitis associated with organized CSH. The symptoms and findings at the time of admission improved without any treatment, blood culture results on day 2 were sterile, and CSF could not be obtained; therefore, the symptoms were suspected to have occurred because of chronic meningitis.

There are two possible routes of entry: 1) the patient had contracted the *C. insulaenigrae* infection before admission, which was not identified by the microbiological results at the time of admission. 2) He contracted the infection during his stay at home (day 21 to 22); he was readmitted to the hospital on day 22 when he presented with a fever and neurological symptoms. However, he confirmed that he was not in contact with salt water or marine mammals during that time.

We compared the characteristics of all the *C. insulaenigrae* strains reported until date (Table 4). The strains isolated from humans were able to grow at 42 °C, while the strains isolated from marine mammals were unable to grow at this temperature. In addition, the gene sequences for the 16S rRNA, obtained from GenBank were different between the strains isolated from humans and the strains isolated from marine mammals. This suggested that there are multiple genotypes of the bacterial strain, which could correspond to the different hosts or infection sites. Considering that the isolate from the current study caused infection in an immunocompetent patient, the difference in genotypes could be related to the differences in the pathogenicity of the bacterial strain.

**Table 4:** Characteristics of *Campylobacter insulaenigrae* isolated from marine mammals and humans

Characteristics	Foster G et al. (2004) [1]	Chua K et al. (2007) [4]	Patient in this study(2015)
Host	Marine mammal	human	human
Growth at/in:			
25 °C	–	–	
42 °C	–	+	+
1% glycine	+	–	
2% NaCl	–	–	+
Oxidase	+	+	+
Hippurate hydrolysis	–	–	–
Cephalothin	–	–	–
Nalidixic acid	–	–	–
Microaerophilic growth	+	+	+
Anaerobic growth	–	–	–
GenBank	AJ620504	EF433401	DQ174183

The growth characteristics of the *C. insulaenigrae* strains reported till date. The strains isolated from humans and marine mammals have differences in growing temperature and genotypes. These factors might relate to the pathogenicity or infection sites

TOnly two cases of *C. insulaenigrae* infection in humans have been reported so far, to the best of our knowledge; however, there is a possibility that *C. insulaenigrae* infection was reported as *C. jejuni* infection, because of the absence of sodium hippurate hydrolysis in both the strains. Whole genome sequencing of the two *C. insulaenigrae* strains might help uncover the reasons behind the differences in the infection sites and infection manifestation between these two strains. Further cases are needed to identify the differences in the characteristics of the bacterial strains arising from the different genotypes.

## Data Availability

All data generated or analyzed during this study are included in this published article.

## Abbreviations

CSF: Cerebrospinal fluid
CSH: Chronic subdural hematoma
MRI: Magnetic resonance imaging
MMT: Manual muscle test
CT: Computed tomography
WBC: White blood cell
CRP: C-reactive protein
